# Minimal percolating sets for mutating infectious diseases

**DOI:** 10.1103/PhysRevResearch.2.023001

**Published:** 2020-04-01

**Authors:** Yuyuan Luo, Laura P. Schaposnik

**Affiliations:** ^1^ Central High School, Grand Rapids, Michigan 49546, USA; ^2^ Department of Mathematics, Statistics and Computer Science, University of Illinois, Chicago, Illinois 60607, USA; ^3^ Mathematical Sciences Research Institute, Berkeley, California 94720, USA

## Abstract

This paper is dedicated to the study of the interaction between dynamical systems and percolation models, with views toward the study of viral infections whose virus mutate with time. Recall that r-bootstrap percolation describes a deterministic process where vertices of a graph are infected once r neighbors of it are infected. We generalize this by introducing *F(t)-bootstrap percolation*, a time-dependent process where the number of neighboring vertices that need to be infected for a disease to be transmitted is determined by a percolation function F(t) at each time t. After studying some of the basic properties of the model, we consider smallest percolating sets and construct a polynomial-timed algorithm to find one smallest minimal percolating set on finite trees for certain F(t)-bootstrap percolation models.

## I. INTRODUCTION

The study of infectious diseases though mathematical models dates back to 1766, when Bernoulli developed a model to examine the mortality due to smallpox in England [1]. Moreover, the germ theory that describes the spreading of infectious diseases was first established in 1840 by Henle and was further developed in the late 19th and early 20th centuries. This laid the groundwork for mathematical models as it explained the way that infectious diseases spread, which led to the rise of compartmental models. These models divide populations into compartments (also called coarse-grained models), where individuals in each compartment have the same characteristics; Ross first established one such model in 1911 in Ref. [2] to study malaria and later on, basic compartmental models to study infectious diseases were established in a sequence of three papers by Kermack and McKendrick [3] (see also Ref. [4]).

In this paper we are interested in the interaction between dynamical systems and percolation models, from the point of view of infections which mutate with time. The use of stochastic models to study infectious diseases has been popular for a long time, and dates back to the 1970s (e.g., see the celebrated work of Harris [5] and Metz [4]). There are many ways to mathematically model infections, including statistics-based models such as regression models (e.g., Ref. [6]), cumulative sum charts (e.g., Ref. [7]), hidden Markov models (e.g., Ref. [8]), and spatial models (e.g., Ref. [7]), as well as mechanistic state-space models such as continuum models which are described by differential equations (e.g., Ref. [9]), stochastic models (e.g., Ref. [10]), complex network models (e.g., Ref. [11]), and agent-based simulations (e.g., Ref. [12]—see also Ref. [1] and references therein).

Difficulties when modeling infections include incorporating the dynamics of behavior in models, as it may be difficult to access the extent to which behaviors should be modeled explicitly, quantifying changes in reporting behavior, as well as identifying the role of movement and travel [13]. When using data from multiple sources, difficulties may arise when determining how the evidence should be weighted and when handling dependence between datasets [14].

In what follows we shall introduce a novel type of dynamical percolation which we call *F(t)-bootstrap percolation*, thought of as a generalization of classical bootstrap percolation. This approach allows us to model mutating infections, and thus we dedicate this paper to the study of some of its main features. After recalling classical r-bootstrap percolation in Sec. I A, we introduce a time-dependent percolation function F(t) through which we introduce a dynamical aspect for the percolating model, as described in Definition 1 in Sec. II, given as follows. Given a function F(t):N→N, we define an *F(t)-bootstrap percolation model* on a graph G with vertices V and initially infected set $A_{0}$ as the process which at time t+1 has infected set given by

(1) $$\begin{array}{c} A_{t+1}=A_{t}\cup \{v\in V:|N\left(v\right)\cap A_{t}\left|\geq F\left(t\right)\right\}, \end{array}$$

where N(v) denotes the set of neighboring vertices to v, and we let $A_{\infty }$ be the final set of infected vertices once the percolation process has finished.

As mentioned before, this model allows one to study situations in which an infection propagates at different rates depending on the time. As an example one may consider the case of the mutating virus of influenza within this setting: instead of having a mutating virus for which the current vaccination becomes ineffective for the new mutation, we can think of the setting as a model with a fixed virus for which it is the rate of infection which is the one that changes with time—in this case, the rate becomes higher as time passes.

In Sec. II we study some basic properties of this model, describe certain (recurrent) functions which ensure the model percolates, and study the critical probability $p_{c}$. Since our motivation comes partially from the study of effective vaccination programs which would allow to contain an epidemic, we are interested both in the percolation time of the model, as well as in minimal percolating sets. We study the former in Sec. III, where by considering equivalent functions to F(t), we obtained bounds on the percolating time.

Finally, in Secs. IV and V we introduce and study smallest minimal percolating sets for F(t)-bootstrap percolation on (nonregular) trees. This leads to one of our main results in Sec. V D, where we describe an algorithm for finding the smallest minimal percolating sets. Last, we conclude the paper with a comparison in Sec. VII between our model and our algorithm for this model with the one considered in Ref. [15] that solves the same problem for classical bootstrap percolation, and analyze the effect of taking different functions within our dynamical percolation.

### A. Bootstrap percolation

The model introduced in this paper, described in Eq. (1), is a dynamical generalization of what is known as *bootstrap percolation*, introduced in 1979 in the context of solid state physics to analyze diluted magnetic systems in which strong competition exists between exchange and crystal-field interactions [16]. Bootstrap percolation has seen applications in diverse areas, including the studies of fluid flow in porous areas, the orientational ordering process of magnetic alloys, as well as the failure of units in a structured collection of computer memory (e.g., see Ref. [17]).

Bootstrap percolation has long been studied mathematically on arbitrary trees [18], as well as on finite and infinite rooted trees including Galton-Watson trees (e.g., see Ref. [19]). Compared with other models for infectious diseases, cellular automata models better simulate the effects of individual behavior and the spatial aspects of epidemic spreading, and better account for the effects of mixing patterns of individuals, as each individual is modeled separately, instead of all individuals being assumed as homogeneous. Hence, contagious diseases in which these factors have significant effects are better understood when analyzed with cellular automata models such as bootstrap percolation [20], which is defined as follows. For $n\in Z^{+}$, we define an *r-bootstrap percolation model* on a graph G with vertices V and initially infected set $A_{0}$ as the process in which at time t+1 has infected set given by

(2) $$\begin{array}{c} A_{t+1}=A_{t}\cup \{v\in V:|N\left(v\right)\cap A_{t}\left|\geq r\right\}. \end{array}$$

Here, as before, we denoted by N(v) the set of neighboring vertices to v.

In contrast, a *SIR Model* relates at each time t the number of susceptible individuals S(t) to the number of infected individuals I(t) and the number of recovered individuals R(t), by a system of differential equations—an example of a SIR model used to simulate the spread of the dengue fever disease appears in Ref. [21]. In these models, a fixed parameter β denotes the average number of transmissions from an infected node per time period. In particular, the rate of spread of diseases are not necessarily constant in these models. This helps motivate the introduction of a time-dependent model of bootstrap percolation where the rate of spread varies according to time, done in Sec. II.

**FIG. 1.**
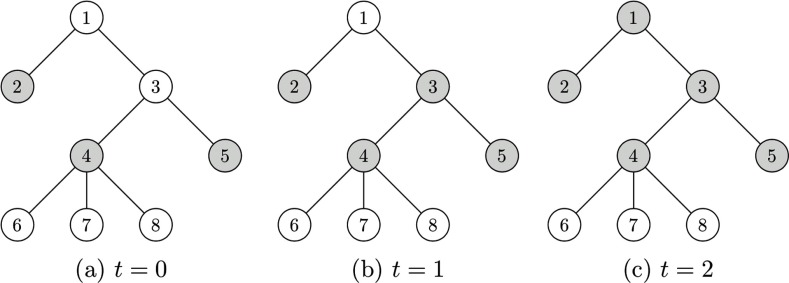
Depiction of 2-bootstrap percolation, where shaded vertices indicated infected nodes.

In what follows we shall present a dynamical generalization of the above model, for which it will be useful to have an example to establish the comparisons. Consider the (irregular) tree with three infected nodes at time t=0, given by $A_{0}=\left\{2,4,5\right\}$ as shown in Fig. 1. Through 2-bootstrap percolation at time t=1, node 3 becomes infected because its neighbors 4 and 5 are infected at time t=0. At time t=2, node 1 becomes infected since its neighbors 2 and 3 are infected at time t=1. Finally, note that nodes 6,7,8 cannot become infected because they each have only 1 neighbor, yet two or more infected neighbors are required to become infected.

## II. TIME-DEPENDENT PERCOLATION

The motivation of time-dependent percolation models appears since the rate of spread of diseases may change over time. In the SIR models mentioned before, since β is the average number of transmissions from an infected node in a time period, 1/β is the time it takes to infect a node. If we “divide the work” among several neighbors, then 1/β is also the number of infected neighbors needed to infect the current node.

Consider now an infection which would evolve with time. This is, instead of taking the same number of neighbours in r-bootstrap percolation, consider a percolation model where the number of neighbours required to be infected for the disease to propagate changes with time, following the behavior of a function F(t). We shall say a function is a *percolation function* if it is a function F:I→N where I is an initial segment of N (or all of N) that we use in a time-dependent percolation process, and which specifies the number of neighbors required to infect a node at time t.

Definition 1 (F(t)-Bootstrap percolation). Given a function F(t):N→N, we define an *F(t)-bootstrap percolation model* on a graph G with vertices V and initially infected set $A_{0}$ as the process in which at time t+1 has infected set given by

(3) $$\begin{array}{c} A_{t+1}=A_{t}\cup \{v\in V:|N\left(v\right)\cap A_{t}\left|\geq F\left(t\right)\right\}. \end{array}$$

Here, as before, we denoted by N(v) the set of neighboring vertices to v, and we let $A_{\infty }$ be the final set of infected vertices once the percolation process has finished.

One should note that r-bootstrap percolation can be recovered from F(t)-bootstrap percolation by setting the percolation function to be the constant F(t)=r. In what follows, unless otherwise stated, the initial set $A_{0}$ is chosen in the same way as in r-bootstrap percolation: by randomly selecting a set of initially infected vertices with probability p, for some fixed value of p which is called the *probability of infection*.

If there are multiple percolation functions and initially infected sets under consideration, then we may use the notation $A_{t}^{F}$ to denote the set of infected nodes at time t percolating under the function F(t) with $A_{0}$ as the initially infected set. In particular, this would be the case when generalising the above dynamical model to a multitype bootstrap percolation model such as the one introduced in Ref. [22].

To understand some basic properties of F(t)-bootstrap percolation, we shall first focus on a single update function F(t), and consider the critical probability $p_{c}$ of infection for which the probability of percolation is $\frac{1}{2}$ on finite trees—in the case of infinite trees, this is the value below which there are no clusters, and above which there are infinite clusters, with probability 1. When considering classical bootstrap percolation, note that the resulting set $A_{\infty }^{r}$ of r-bootstrap percolation is always contained in the resulting set $A_{\infty }^{n}$ of n-bootstrap percolation provided n≤r. From the above, setting the value $m:=\min_{t}F\left(t\right)$, the resulting $A_{\infty }^{F}$ set of F(t)-bootstrap percolation will be contained in $A_{\infty }^{m}$.

Note that for any time $t_{★}$ such that

$$F(t_{★})=m,$$

one has that if $v\in A_{t_{★}}^{F}$, then $v\in A_{t_{★}^{\prime }}^{F}$ for the next time $t_{★}^{\prime }$ for which $F(t_{★}^{\prime })=m$. Moreover, since for the recurrent functions we are considering there are infinitely many times $t_{★}$ such that $F(t_{★})=m$, one has that the final resulting set $A_{\infty }^{m}$ of m-bootstrap percolation is contained in the final resulting set $A_{\infty }^{F}$ of F(t)-bootstrap percolation. Then, the resulting sets of m-bootstrap percolation and F(t)-bootstrap percolation need to be identical, and hence the critical probability for F(t)-bootstrap percolation is that of m-bootstrap percolation. In other words, we have shown that if F(t) equals its minimum for infinitely many times t, then the critical probability of infection $p_{c}$ for which the probability of percolation is 1/2, is given by the value of the critical probability in m-bootstrap percolation for $m:=\min_{t}F\left(t\right)$, this is

(4) $$\begin{array}{ccc} p_{c}\left[F\left(t\right)-\text{bootstrap}\right] & = & p_{c}\left(m-\text{bootstrap}\right)\\ \mathrm{for}\;m & = & \min_{t}F\left(t\right). \end{array}$$

The type of update functions that satisfy this include sinusoidal functions and, since we restricted the codomain to be positive, weakly decreasing functions.

The percolation function F(t) can be written in terms of a one-parameter family of parameters β by setting $F\left(t\right):=\left\lceil \frac{1}{\beta (t)}\right\rceil$. As we shall see later, different choices of the one-parameter family β(t) defining F(t) will lead to very different dynamical models. A particular setup arises from Ref. [23], which provides data on the time-dependent rate of a specific virus spread, and through which one has that an interesting family of parameters appears by setting

$$\beta \left(t\right)=\left(b_{0}-b_{f}\right){\left(1-k\right)}^{t}+b_{f},$$

where $b_{0}$ is the initial rate of spread, $b_{f}$ is the final rate of spread, and 0<k<1. Then at time t, the number of infected neighbors it takes to infect a node is

$$F\left(t\right):=\left\lceil \frac{1}{\left(b_{0}-b_{f}\right)\cdot {\left(1-k\right)}^{t}+b_{f}}\right\rceil .$$

In this case, since β(t) tends to $b_{f}$, and $\frac{1}{\beta }$ tends to $\frac{1}{b_{f}}$, one cans see that there will be infinitely many times t such that

$$F\left(t\right)=\left\lceil \frac{1}{b_{f}}\right\rceil .$$

Hence, in this setting from Eq. (4), the critical probability will be same as that of a r-bootstrap percolation where $r=\lceil \frac{1}{b_{f}}\rceil$.

## III. PERCOLATION TIME

Informally, the *percolation time* (for finite graphs) is the time it takes for the percolation process to terminate, starting from a specific initially infected set of a graph. In terms of limits, recall that the final percolating set is defined as

(5) $$\begin{array}{c} A_{\infty }:=\lim_{t\to \infty }A_{t}, \end{array}$$

and thus one may think of the percolation time as the smallest time t for which $A_{t}=A_{\infty }$. Note that for percolation on all infinite trees, there exists a percolation function and an initially infected set such that a percolation time does not exist, whereas there is always a defined percolation time for percolation on finite trees. Thus, we restrict our following discussions to finite trees.

**FIG. 2.**
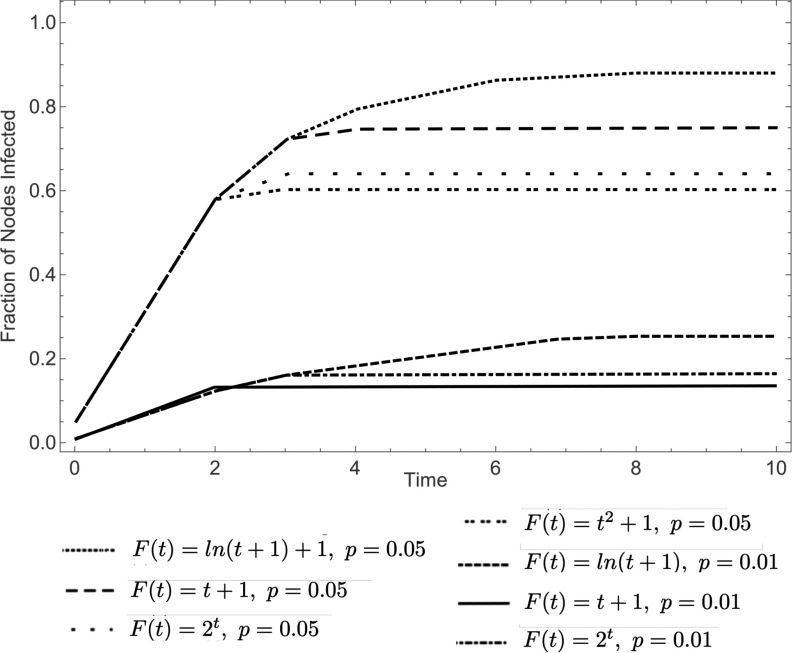
Percentage of nodes infected at time t for F(t)-bootstrap percolation with initial probability p, on graphs with 100 nodes and 300 edges.

By considering different initial probabilities of infection p which determine the initially infected set $A_{0}$, and different percolation functions F(t) one can see that the percolation time of a model can vary drastically. To illustrate this, in Fig. 2 we have plotted the percentage of nodes infected with two different initial probabilities and four different percolation functions. The model was ran $10^{3}$ times for each combination on random graphs with $10^{2}$ nodes and 300 edges.

In the settings of Fig. 2, one can see that all the models stabilize by time 10, implying that the percolation time is less than or equal to 10. Generally, understanding the percolation time is useful in determining when the disease spreading has stabilized. In what follows, we find a method to generate an upper bound on the percolation time given a specific graph and function. Formally, for the percolation functions considered in this paper, we define the *percolation time* $t_{*}$ as the minimum

$$t_{*}:=\min_{t}\left\{t|A_{t+1}=A_{t}\right\}.$$

### A. Equivalent functions

Expanding on the notation of Eq. (5), we shall denote by $A_{\infty }^{F}$ the set of nodes infected by percolating the set $A_{0}$ on the graph with percolation function F(t), and we shall simply write $A_{\infty }$ when the percolation function F(t) is clear from context or irrelevant. Moreover, we shall say that two percolation functions $F_{1}:I_{1}\to Z^{+}$ and $F_{2}:I_{2}\to Z^{+}$ are *equivalent* (written as $F_{1}\equiv F_{2})$ for the graph G if for all initially infected sets $A_{0}$, one has that

(6) $$\begin{array}{c} A_{\infty }^{F_{1}}=A_{\infty }^{F_{2}}. \end{array}$$

This equivalence relation can be understood through the lemma below, which uses an additional function γ(t) to relate two percolation functions $F_{0}$ and $F_{0}^{\prime }$ if $F_{0}^{\prime }$ can be intuitively “generated” by removing some values of $F_{0}$. This removal procedure is further specified below.

Given two subsets $I_{1}$ and $I_{2}$ of N, we say a function $\gamma :I_{1}\to I_{2}\cup \left\{-1\right\}$ is a *nice function* if it is surjective and

1. it is injective on $\gamma^{-1}\left(I_{2}\right)$;
2. it is increasing on $\gamma^{-1}\left(I_{2}\right)$;
3. it satisfies γ(a)≤a or γ(a)=−1.

The notion of a nice function allows us to understand the relation between two different dynamical percolation models defined through two functions F(t) and $F^{\prime }\left(t\right)$. Given $I_{1},I_{2}\subset N$, let F(t) be any percolation function with domain $I_{1}$, and define the percolation function $F^{\prime }\left(t\right)$ with domain $I_{2}$ as

$$F^{\prime }\left(t\right):=F\left[\gamma^{-1}\left(t\right)\right]$$

for γ(t) a nice function. Through the function $F^{\prime }\left(t\right)$, for any fixed initially infected set $A_{0}$ and $t\in I_{2}$, one can show by induction (see Appendix A) that

(7) $$\begin{array}{c} A_{t}^{F^{\prime }}\subseteq A_{\gamma^{-1}\left(t\right)}^{F}. \end{array}$$

Intuitively, the above results tell us that given a fixed time $t_{0}$ and some $t>t_{0}$, if F(t)=ℓ is the smallest value the function takes on after the time $t_{0}$, and F(t) has already taken on that value more than ℓ times, for ℓ the number of nodes in the graph, then there will be no nodes that will be infected at that time and the value is safe to be “removed.”

### B. Removal process

In what follows we shall clarify the removal process, by defining an upper bound on percolation time on a specified tree and function F(t). For this, let G be a regular tree of degree d and ℓ vertices. Given a percolation function F(t), define the functions $F^{\prime }\left(t\right)$ and γ:N→N∪{−1} by setting:

(i) $F^{\prime }\left(0\right):=F\left(0\right)$, and γ(0):=0.
(ii) Suppose the least value we have not considered F(t) at is a, and let b be the least value where $F^{\prime }\left(b\right)$ has not yet been defined. If F(a) has not yet appeared ℓ times since the last time t such that F(t)<F(a) and F(a)≤d, then set $F^{\prime }\left(b\right):=F\left(a\right)$, and let γ(a)=b. Otherwise, γ(a)=−1.

Then, one can show (see Appendix B) that the two functions are equivalent as defined in Eq. (6), this is

(8) $$\begin{array}{c} F^{\prime }\left(t\right)\equiv F\left(t\right). \end{array}$$

From the above description of equivalent functions, we can see two things:

(i) The upper bound on the percolation time is the time of the largest t such that $F^{\prime }\left(t\right)$ is defined, and we can use this function in an algorithm to find the smallest minimal percolating set since F(t) and $F^{\prime }\left(t\right)$ are equivalent.
(ii) An upper bound on the percolation time can not be obtained without regards to the percolation function.

To see item (ii), suppose we have such an upper bound b on some connected graph with degree d and with 1 node initially infected and more than 1 node not initially infected. If we have percolation function F(t) such that F(t)=d+1 for all t∈N≤b and F(m)=1 otherwise, then we see that there will be nodes infected at time b+1, leading to a contradiction.

To see the implications of the above points within the equivalence of functions, suppose that the degree of the graph in consideration is d, and define a sequence a, where $a_{1}=d$ and

$$a_{n+1}=\left(a_{n}+1\right)d.$$

Then, the size of the domain of $F^{\prime }\left(t\right)$ in Eq. (8) is

(9) $$\begin{array}{c} \Sigma_{i=1}^{d}a_{i}. \end{array}$$

Indeed, suppose each value do appear exactly d times after the last value smaller than it appears. To count how large the domain can be, we start with the possible ts such as $F^{\prime }\left(t\right)=1s$ in the function; there are d of them as 1 can maximally appear d times. Note that this is equal to $a_{1}$. Now, suppose we have already counted all the possible ts when $F^{\prime }\left(t\right)<n+1$, for 1leqn<d, which amounted to $a_{n}$. Then, there can be maximally d instances at the between the appearance of each t when $F^{\prime }\left(t\right)<n$ as well as before and after all such appearances, so there are $a_{n}+1$ places where $F^{\prime }\left(t\right)=n$ can appear. Thus, there are maximally $(a_{n}+1)d$ elements t in the domain such that $F^{\prime }\left(t\right)=n+1$. Summing all of them yields $\Sigma_{i=1}^{d}a_{i}$, the total number of elements in the domain in Eq. (9). Finally, note that from Eq. (8), for some F(t), $A_{0}$ and n, one has $A_{\gamma^{-1}\left(n\right)}^{F}=A_{n}^{F^{\prime }}$. If $A_{\infty }^{F^{\prime }}$ is reached by time $\Sigma_{i=1}^{d}a_{i}$, then the set must be infected by time $\gamma^{-1}\left(\Sigma_{i=1}^{d}a_{i}\right)$. Hence, in this setting an upper bound of F(t) percolating on a graph with d vertices can be found by taking $\gamma^{-1}\left(\Sigma_{i=1}^{d}a_{i}\right)$, as defined in Eq. (9).

## IV. MINIMAL PERCOLATING SETS

When considering percolations within a graph, it is of much interest to understand which subsets of vertices, when infected, would lead to the infection reaching the whole graph. To study those sets, we shall refer to a *percolating set* of a graph G with percolation function F(t) is a set $A_{0}$ for which $A_{\infty }^{F}=G$ at a finite time. A *minimal percolating set* is a percolating set A such that if any node is removed from A, it will no longer be a percolating set.

A natural motivation for studying minimal percolating sets is that as long as we keep the number of individuals infected to less than the size of the minimal percolating set, we know that the entire population will not be decimated. Bounds on minimal percolating sets on grids and other less regular graphs have extensively been studied. For instance, it has been shown in Ref. [24] that for a grid ${\left[n\right]}^{d}$, there exist a minimal percolating set of size

$$4n^{2}/33+o\left(n^{2}\right),$$

but there does not exist one larger than ${\left(n+2\right)}^{2}/6$. In the case of trees, Ref. [15] gives an algorithm that finds the largest and smallest minimal percolating sets on trees. Since then, only a few further results have been obtained improving those bounds (see, for example, the work on degree conditions for bootstrap percolation from small sets in Ref. [25] and references therein). However, the results in the above papers cannot be easily extended to the dynamical model because it makes several assumptions such as F(t)≠1 that do not necessarily hold in the dynamical model.

**FIG. 3.**
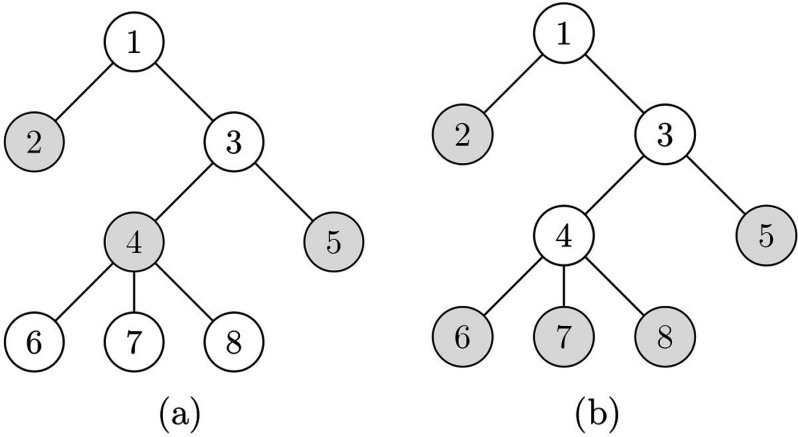
(a) In this tree, having nodes 2,4,5 infected (shaded) initially is sufficient to ensure that the whole tree is infected. (b) This minimal percolating set shaded is of size 5.

In the following sections we shall study minimal percolating sets for certain models of F(t)-bootstrap percolations, but before this is done, we shall first consider an example of a minimal percolating set with F(t)=t, as shown in Fig. 3.

In this case, the minimal percolating set has size 3, as shown in Fig. 3(a). Indeed, we see that if we take away any of the shaded nodes, the remaining initially infected shaded nodes would not percolate to the whole tree, and thus they form a minimal percolating set; further, there exists no minimal percolating sets of size 1 or 2, thus this is the smallest minimal percolating set. It should be noted that minimal percolating sets can have different sizes. For example, another minimal percolating set with 5 vertices appears in Fig. 3(b).

In what follows we shall work with general finite trees T(V,E) with set of vertices V and set of edges E. In particular, we shall consider the smallest minimal percolating sets in the following section.

## V. ALGORITHMS FOR FINDING SMALLEST MINIMAL PERCOLATING SET

Consider F(t)-bootstrap percolation on a tree T(V,E) with initially infected set $A_{0}\subset V$. As before, we shall denote by $A_{t}$ be the set of nodes infected at time t. For simplicity, we shall use here the word “filled” synonymously with “infected.”

### A. Smallest and largest times

To build an algorithm to find smallest percolating sets, we first need to introduce a few definitions that will simplify the notation at later stages. First, we shall denote by L(a) the largest time t such that a≤F(t), and if there does not exist such a time t, then set L(a)=∞, this is

(10) $$\begin{array}{c} L\left(a\right)=\left\{\begin{array}{cc} \max_{t}\left\{t\;:\;a\leq F\left(t\right)\right\} & \mathrm{if}\;\mathrm{it}\;\mathrm{exists},\\ \infty & \mathrm{otherwise}. \end{array}\right. \end{array}$$

Similarly, let B(a) be the smallest time t such that a≤F(t), and if such a time t does not exist, set B(a)=∞, leading to

(11) $$\begin{array}{c} B\left(a\right)=\left\{\begin{array}{cc} \min_{t}\left\{t\;:\;a\leq F\left(t\right)\right\} & \mathrm{if}\;\mathrm{it}\;\mathrm{exists},\\ \infty & \mathrm{otherwise}. \end{array}\right. \end{array}$$

Given a,b∈N, if a<b, then L(a)≥L(b). Indeed, this holds because if a node can be infected to with b neighbors, then it can with a neighbors where a<b. Note that in general, a smallest percolating set $A_{0}$ must be a minimal percolating set. To see this, suppose not. Then there exists some v in $A_{0}$ such that $A_{0}-\left\{v\right\}$ percolates the graph. That means that $A_{0}-\left\{v\right\}$, a smaller set that $A_{0}$, is a percolating set. However, since $A_{0}$ is a smallest percolating set, we have a contradiction. Hence, showing that a percolating set $A_{0}$ is the smallest implies that $A_{0}$ is a minimal percolating set.

The first algorithm one may think of is to try every case. There are $2^{n}$ possible sets $A_{0}$, and for each set we much percolate $A_{0}$ on T to find the smallest percolating set. This amounts to an algorithm of complexity,

$$O(t2^{n}),$$

where t is the upper bound on the percolation time. In what follows we shall describe a polynomial-timed algorithm to find the smallest minimal percolating set on T(V,E), described in the algorithm. For this, we shall introduce two particular times associated to each vertex in the graph, and formally define what isolated vertices are.

### B. Isolated nodes

For each node v in the graph, we let $t_{a}\left(v\right)$ be the time when it is infected, and $t_{*}\left(v\right)$ the time when it is last allowed to be infected; moreover, when building our algorithm, each vertex will be allocated a truth value of whether it needs to be further considered. A node v is said to be *isolated* with regards to $A_{0}$ if there is no vertex w∈V such that v becomes infected when considering F(t)-bootstrap percolation with initial set $A_{0}\cup \left\{w\right\}$. From these definitions, a node is isolated with regards to a set if it is impossible to infect it by adding one of any other node to that set that is not itself.

Building toward the percolating algorithm, we shall show a few properties first. First, note that if a node cannot be infected by including a neighbor in the initial set, it is isolated. Hence, by filling the neighbor in the initial set, we either increased the number of neighbors infected to a sufficient amount, or we expanded the time allowed to percolate with fewer neighbors so that percolation is possible.

A quick test to see whether a vertex is isolated can be done as follows. Let v be an uninfected node such that not all of its n neighbors are in set $A_{0}$. Define a function

(12) $$\begin{array}{c} N:\{0,1,...,n\}\to Z, \end{array}$$

where N(i) is the smallest time when i of the neighbors of node v is infected, and set N(0)=0. Then, a vertex v is isolated iff there exists no i such that

(13) $$\begin{array}{c} F\left(t\right)\leq i+1\;\mathrm{for}\;\mathrm{some}\;t\in (N\left(i\right),t_{*}]. \end{array}$$

To see that this test works, suppose $s\in N\left(v\right)\cap A_{0}$. If there exists i such that F(t)≤i+1 for some $t\in (N\left(i\right),t_{*}]$, then using $A_{0}\cup \left\{s\right\}$ as the initially infected set allows percolation to happen at time t since there would be i+1 neighbors infected at each time N(i). Thus, by contradiction, the forward direction is proven.

Let v be not isolated, and $v\in P(A_{0}\cup \left\{s\right\})$ for some neighbor s of v. Then there would be i+1 neighbors infected at each time N(i). Moreover, for v being to be infected, the i+1 neighbors must be able to fill v in the allowed time, $\left(N\left(i\right),t_{*}\right]$. Thus, there exists N(i) such that F(t)≤i+1 for some $t\in (N\left(i\right),t_{*}]$. By contradiction, we proved the backwards direction.

### C. Variation of initial sets

Note that if a vertex v is uninfected and $N\left(v\right)\subset A_{0}$, then the vertex must be isolated. In what follows we shall study the effect of having different initially infected sets when studying F(t)-bootstrap percolation. For this, let Q be an initial set for which a fixed vertex v with n neighbours is isolated. Denoting the neighbors of v be $s_{1},s_{2},...,s_{n}$, we let the times at which they are infected be $t_{1}^{Q},t_{2}^{Q},...,t_{n}^{Q}$. Here, if for some 1≤i≤n, the vertex $s_{i}$ is not infected, then set $t_{i}^{Q}$ to be some arbitrarily large number. Moreover, consider another initial set P such that the times at which $s_{1},s_{2},...,s_{n}$ are infected are $t_{1}^{P},t_{2}^{P},...,t_{n}^{P}$ satisfying

(14) $$\begin{array}{ccc} t_{i}^{Q}= & t_{i}^{P} & \;\mathrm{for}\;i\neq j, \end{array}$$

(15) $$\begin{array}{ccc} t_{j}^{Q}\leq & t_{j}^{P} & \;\mathrm{for}\;i=j, \end{array}$$

for some 1≤j≤n.

In the above setting, if v∉P, then the vertex v must be isolated with regards to P as well. Indeed, consider $N_{Q}\left(i\right)$ as defined in Eq. (12) for the set Q, and $N_{P}\left(i\right)$ the corresponding function for the set P. Then for all integers k∈{0,1,...,n}, one has that $N_{Q}\left(k\right)\leq N_{P}\left(k\right)$. Indeed, this is because with set P, each neighbor of v is infected at or after they are with set Q. Then, from Eq. (3), v is isolated with regards to Q so there is no m such that

$$F\left(t\right)\leq m+1\;\text{for}\;\text{some}\;t\in (N_{Q}\left(m\right),t_{*}].$$

However, since

$$N_{Q}\left(k\right)\leq N_{P}\left(k\right)\;\text{for}\;\text{all}\;k\in \left\{0,1,...,n\right\},$$

we can say that there is no m such that

$$F\left(t\right)\leq m+1\;\text{for}\;\text{some}\;t\in (N_{P}\left(m\right),t_{*}],$$

as $\left(N_{P}\left(m\right),t_{*}\right]\subseteq \left(N_{Q}\left(m\right),t_{*}\right].$ Thus, we know that v must also be isolated with regards to P.

Given a vertex v which is not isolated with n infected neighbors, we shall define $t_{p}\left(v\right)\in \left(0,t_{*}\right]$ to be the largest integer such that for i∈{0,1,...n}, one has that

(16) $$\begin{array}{c} F(t_{p})\leq i+1. \end{array}$$

Note that to fill an isolated node v, one can fill it by filling one of its neighbors by time $t_{p}\left(v\right)$, or just add the vertex it to the initial set. Hence, one needs to fill a node $v_{n}$ which is either the parent $\mathrm{par}(v_{n})$, a child $\mathrm{chi}(v_{n})$, or itself.

One can further understand the variation of initially infected sets by noting that, given an isolated node $v\notin A_{0}$, to achieve percolation, it is always better (faster) to include v in $A_{0}$ than attempting to make v unisolated. Indeed, it is possible to make v isolated by including only descendants of v in $A_{0}$ since we must include less than deg(v) neighbors. But we know that if given the choice to include a descendant or a v to the initial set, choosing v is absolutely advantageous because the upwards percolation achieved by v infected at some positive time is a subset of upwards percolation achieved by filling it at time 0. Thus, including v to the initial set is superior.

The above set-up can be understood further to find which vertex needs to be chosen to be $v_{n}$. To see this, consider a vertex $v\notin A_{0}$. Then, in finding a node u to add to $A_{0}$ so that $v\in A_{\infty }$ for the initial set $A_{0}\cup \left\{u\right\}$ and such $A_{\infty }$ is maximized, the vertex $v_{n}$ must be the parent par(v) of v. This can be understood by noting that filling v by time $t_{*}\left(v\right)$ already ensures that all descendants of v will be infected, and that all percolation upwards must go through the parent par(v) of v. This means that filling any child of v to fill v (by including some descendant of v in $A_{0}$) we obtain a subset of percolation if we include the parent par(v) of v in $A_{0}$. Therefore, the parent par(v) of v or a further ancestor needs to be included in $A_{0}$, which means $v_{n}$ needs to be the parent par(v) of v.

### D. Smallest minimal percolating set algorithm

Note that given a node $v\notin A_{0}$, if we fill its parent par(v) before $t_{p}\left(v\right)$, then the vertex will be infected. We are now ready for our main result, which improves the naive $O(t2^{n})$ bound for finding minimal percolating sets to O(tn), as discussed further in the last section.

To obtain one smallest minimal percolating set of a tree T(V,E) with percolation function F(t), proceed as follows:

1. Step 1. Initialize tree: For each node v, set $t_{*}\left(v\right)$ to be some arbitrarily large number, and set it to true for needing to be considered.
2. Step 2. Percolate using current $A_{0}$. Save the time $t_{a}$'s at which the nodes were infected. Stop the algorithm if the set of nodes that are infected equals the set V.
3. Step 3. Consider a node v that is furthest away from the root, and if there are multiple such nodes, then choose the one that is isolated, if it exists.

(a) if v is isolated or is the root, then add v to $A_{0}$.
(b) otherwise, set
  $$t_{*}\left[\mathrm{par}\left(v\right)\right]=t_{p}\left(v\right)-1$$
  if it is smaller than the current $t_{*}\left[\mathrm{par}\left(v\right)\right]$ of the parent [for $t_{p}\left(v\right)$ defined in Eq. (16)].
Set v as considered.
4. Step 4. Go to step 2.

After the process has finished, the resulting set $A_{0}$ is one of the smallest minimal percolating sets.

Note that the specification that the tree must be finite is important as the algorithm is iterative and relies on the existence of a node furthest from the root.

The description of the algorithm through which one can find a smallest percolating set, shall be organized as follows: we will first show that the set $A_{0}$ constructed through the steps of the algorithm is a minimal percolating set, and then show that it is the smallest such set. To see that $A_{0}$ is a minimal percolating set, we first need to show that $A_{0}$ percolates. In step 3, we have included all isolated nodes, as well as the root if it wasn't infected already, in $A_{0}$ and guaranteed to fill all other nodes by guaranteeing that their parents will be infected by their time $t_{p}$.

Showing that $A_{0}$ is a minimal percolating set is equivalent to showing that if we remove any node from $A_{0}$, it will not percolate to the whole tree. Note that in the process, we have only included isolated nodes in $A_{0}$ other than the root. This means that if any node $v_{0}$ is removed from $A_{0}$, it will not percolate to $v_{0}$ because we only fill nodes higher than $v_{0}$ after considering $v_{0}$ and since turning a node isolated requires filling at least one node higher and one descendant of $v_{0}$, it cannot be infected to after removing it from $A_{0}$. Moreover, if the root is in $A_{0}$, since we considered the root last, it is implied that the rest of $A_{0}$ does not percolate to the root. Thus, $A_{0}$ is a minimal percolating set.

Now we show that the set $A_{0}$ constructed through the algorithm is of the smallest percolating size by contradiction using Lemma 15. For this, suppose there is some other minimal percolating set B for which |B|≤|A|. Then, we can build an injection $A_{0}$ to B in the following manner: iteratively consider the node a that is furthest from the root and $a\in A_{0}$ that hasn't been considered, and map it to a vertex $b_{0}$ which is itself or one of its descendants of b where b∈B. We know that such a $b_{0}$ must exist by induction.

We first consider the case where a has no descendant in A. Then, if the vertex b∈B and b is a descendant of a, we map a to b. Now suppose there is no node b that is a descendant of a where b∈B. Then, a∈B because otherwise a would be isolated with regards to B as well, by Lemma 15. This means that we can map a to a in this case.

Now we can consider the case where all the descendants d of a such that $d\in A:=A_{0}$ has been mapped to a node $b_{d}\in B$ where $b_{d}$ is d or a descendant of d. If there is such a b∈B, then b is a descendant of a, and thus no nodes in A have been matched to b yet, allowing us to map a to b. Now suppose there is no such b∈B. This means that there is no b∈B such that all of the descendants of a are descendants of b. Then, all nodes in B that are descendants of a is either some descendant of a∈A or some descendant of a descendant of a in A. This means that percolating B, the children of a will all be infected at later times than when percolating A, and by Lemma 15, one has that a∈B because a would be isolated with regards to B. So in this case, we can map a to a.

The map constructed above is injective because each element of B has been mapped to not more than once. Since we constructed an injective function from the set generated by the algorithm $A_{0}$ to a smaller minimal percolating set $B_{0}$, we have a contradiction because $A_{0}$ then must be the same size or larger than $B_{0}$. Thus, the set generated from the algorithm must be a smallest minimal percolating set.

From Sec. V D one can find the smallest minimal percolating set on any finite tree. Moreover, it gives an intuition for how to think of the vertices of the graph: in particular, the property of “isolated” is not an absolute property, but a property relative to the set of nodes that has been infected before it. This isolatedness is easy to define and work with in trees since each node has at most one parent. Moreover, a similar property may be considered in more general graphs and we hope to explore this in future work. Below we shall demonstrate the algorithm of Sec. V D with an example.

### E. Smallest minimal percolating sets on trees

**FIG. 4.**
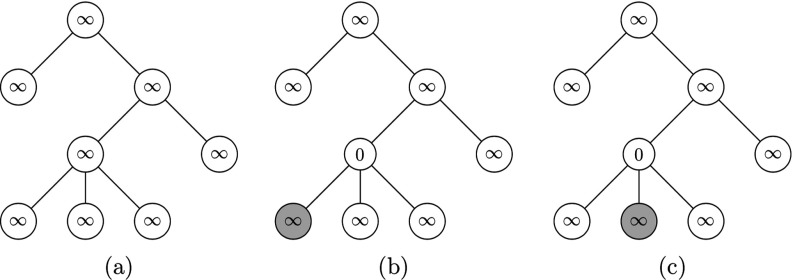
Panels (a–c) show the first three updates through the algorithm in Sec. V D, where the vertices considered at each time are shaded and each vertex is assigned the value of $t_{*}$.

We will preform the algorithm on the tree in Fig. 3, with percolating function F(t)=t. We first initialize all the nodes, setting their time $t_{*}$ to some arbitrarily large number, represented as ∞ in Fig. 4.

Percolating the empty set $A_{0}$, the resulting infected set is empty, as shown in Fig. 4(a). We then consider the furthest node from root. None of them are isolated, so we can consider any; we begin by considering node v=6 in the labeling of Fig. 3. It is not isolated, so we set the value to be

$$t_{*}\left[\mathrm{par}\left(v\right)\right]=t_{p}\left(v\right)-1=0,$$

as can be seen in Fig. 4(b). Then we consider another node furthest from the root, and through the algorithm set the $t_{*}$ of the parent to $t_{p}-1=0$, as can be seen in Fig. 4(c).

**FIG. 5.**
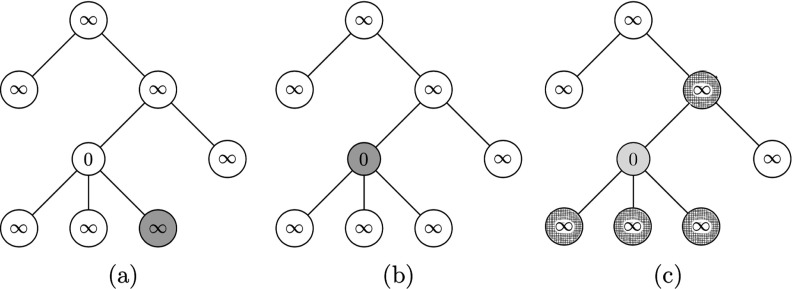
Panels (a, b) update 4–5 through the algorithm. Panel (c) sets $A_{0}$ in light shade, and infected vertices as gridded vertices.

The following steps of the algorithm are depicted in Fig. 5 below. As done in the first three steps of Fig. 4, we consider the next furthest node v from the root, and by the same reasoning as node 6, set the $t_{*}\mathrm{par}\left(v\right)$ of the parent to $t_{*}\mathrm{par}\left(v\right)=1$, as can be seen in Fig. 5(a) below.

**FIG. 6.**
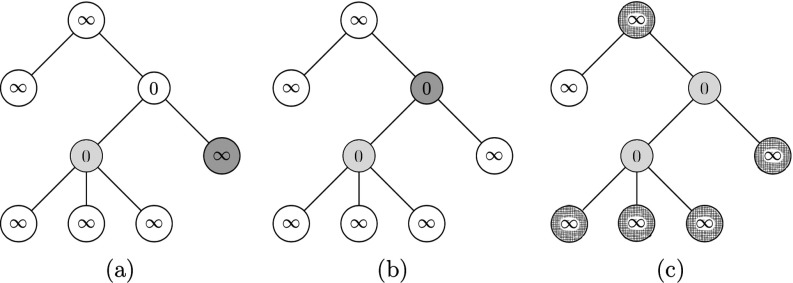
Panels (a–c) update through the algorithm in Sec. V D after setting $A_{0}$ to be as in Fig. 5.

Now we consider node 4: since it is isolated, we fill it in as in Fig. 5(b). The set of nodes infected can be seen in Fig. 5(c). We then consider node 5, the furthest node from the root not considered yet. Since it is not isolated, change the $t_{*}\mathrm{par}\left(v\right)$ of its parent to $t_{p}\left(v\right)-1=0$, as in Fig. 6(a).

**FIG. 7.**
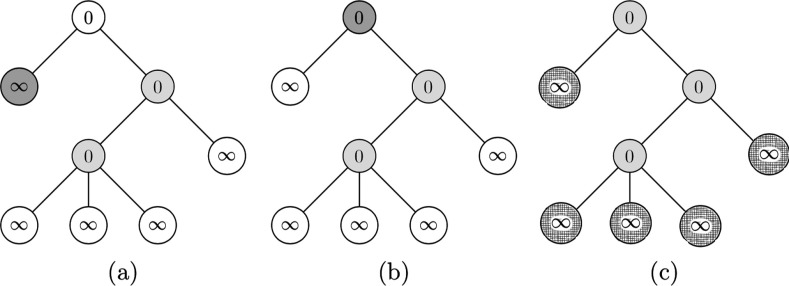
Final steps of the algorithm, as in Fig. 5.

Then we consider node 3, which is isolated, so we include it in $A_{0}$. The infected nodes as a result of percolation by this $A_{0}$ is shown as red vertices in Fig. 6(c). To finish the process, consider the vertex v=2 since it is the furthest away nonconsidered node. It is not isolated so we change the

$$t_{*}\left[\mathrm{par}\left(v\right)\right]=t_{p}\left(v\right)-1=0,$$

as shown in Fig. 7(a). Finally, we consider the root: since it is isolated, we include it in our $A_{0}$ as seen in Fig. 7(b). Finally, percolating this $A_{0}$ results in all nodes being infected as shown in Fig. 7(c), and thus we stop our algorithm.

Through the above algorithm, we have constructed a smallest minimal percolating set shown as red vertices in Fig. 7(c), which is of size 3. Comparing it with Fig. 3, we see that the minimal percolating set in that example is indeed the smallest, also with 3 elements. Finally, it should be noted that in general the times $t_{p}$ for each node could be different from each other and are not the same object.

From the above example, and its comparison with Fig. 3, one can see that a graph can have multiple different smallest minimal percolating sets, and the algorithm finds just one. In the algorithm of Sec. V D, one minimizes the size of a minimal percolating set, relying on the fact that as long as a node is not isolated, one can engineer its parent to become infected so as to infect the initial node. The motivation of the definition of isolated stems from trying to find a variable that describes whether a node is still possible to become infected by infecting its parent. Because the algorithm is on trees, we could define isolation to be the inability to be infected if we add only one node.

## VI. FURTHER PROPERTIES OF F(t)-BOOTSTRAP PERCOLATION AND OUR ALGORITHM

We shall dedicate this section to further the analysis of our algorithm and its complexity, its comparison to the work in Ref. [15], and to consider our model on random trees.

### A. Complexity

First, we shall consider the complexity of the algorithm in Sec. V D to find the smallest minimal percolating set on a graph with n vertices. To calculate this, suppose t is the upper bound on percolation time; we have presented a way to find such an upper bound in the previous sections. In the algorithm, we first initialize the tree, which is linear timed. Steps 2 and 3 are run at most n times as there can only be a total of n unconsidered nodes. The upper bound on time is t, so steps 2 will take t to run. Determining whether a node is isolated is linear timed, so determining isolated-ness of all nodes on the same level is quadratic timed, and doing the specifics of step 3 is constant timed. Thus, the algorithm is

$$O\left[n+n\left(t+n^{2}\right)\right]=O\left(tn+n^{3}\right)=O\left(tn\right),$$

much better than then $O(t2^{n})$ complexity of the naive algorithm.

### B. Comparison on perfect trees

**FIG. 8.**
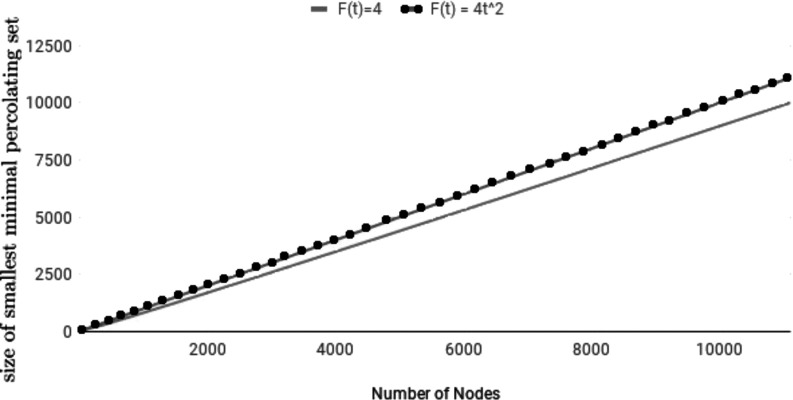
The size of smallest minimal percolating sets on perfect trees with height 4, with a constant and a nonconstant percolation function F(t).

Finally, we shall compare our algorithm with classical r-bootstrap percolation. For this, in Fig. 8 we show a comparison of sizes of the smallest minimal percolating sets on perfect trees of height 4, varying the degree of the tree. Two different functions were compared: one is constant and the other is quadratic. We see that the time-dependent bootstrap percolation model can be superior in modeling diseases with time-variant speed of spread, for that if each individual has around 10 social connections, the smallest number of individuals needed to be infected to percolate the whole population has a difference of around $10^{3}$ between the two models.

### C. Comparison on random trees

**FIG. 9.**
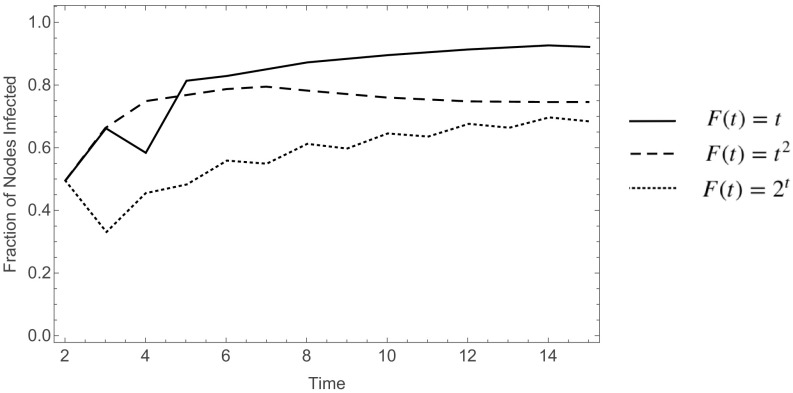
Trials done on 10 000 random trees of n nodes, taking the average, and dividing it by n for the fraction of node needed to be initially infected for the model to percolate.

We shall conclude this work by comparing the smallest minimal percolating sets found through our algorithm and those constructed by Riedl in Ref. [15]. To understand the difference of the two models, we shall first consider in Fig. 9 three percolating functions F(t) on random trees of different sizes, where each random tree has been formed by beginning with one node, and then for each new node i we add, use a random number from 1 to i−1 to determine where to attach this node.

In Fig. 9, the size of the smallest minimal percolating set can be obtained by multiplying the size of the minimal percolating set by the corresponding value of n. In particular, one can see how the exponential function requires an increasingly larger minimal percolating set in comparison with polynomial percolating functions.

### D. Comparison with Ref. [15]

Riedl provided an algorithm for the smallest minimal percolating sets in trees for r-bootstrap percolation in Ref. [15] that runs in linear time. We shall describe his algorithm generally to clarify the comparisons we will make. Riedl defined a trailing star or trailing pseudostar as a subtree with each vertex being of distance at most 1 or 2 away, respectively, from a certain center vertex that is connected to the rest of the tree by only one edge. Then, the first step of Riedl's algorithm is a reduction procedure that ensures every nonleaf has degree at least r: Intuitively, one repeatedly finds a vertex with degree less than r, include it to the minimal percolating set, remove it and all the edges attached to it, and for each of the connected components, add a new node with degree 1 connected to the node that was a neighbor of the node we removed.

**FIG. 10.**
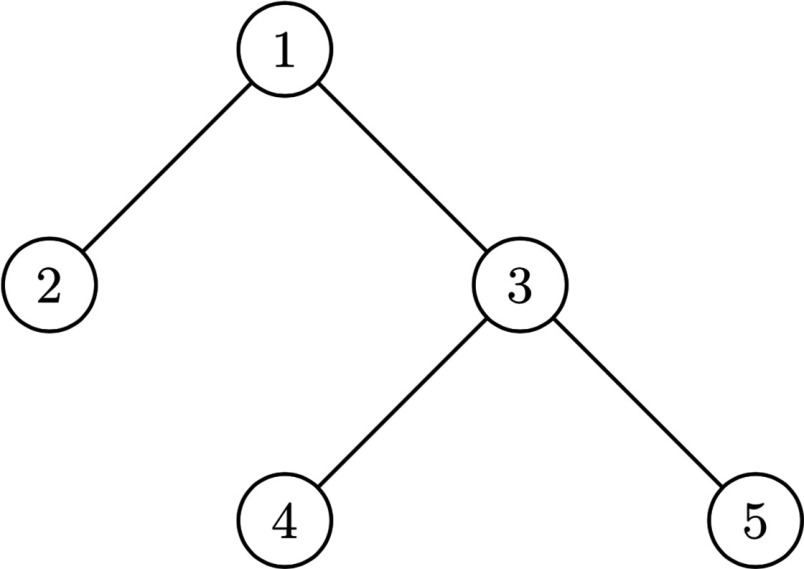
Degree 2 tree with 5 nodes.

Then, the algorithm identifies a trailing star or pseudostar, whose center shall be denoted by v and its set of leaves by L. Letting the original tree be T, if the number of leafs on v is less than r, then set $T^{\prime }=T\setminus \left(v\cup L\right)$; otherwise, set $T^{\prime }=T\setminus L$. Recursively set $A^{\prime }$ as the smallest minimal percolating set of $T^{\prime }$ under r-bootstrap percolation. Then, the smallest minimal percolating set for T is $A^{\prime }\cup L$ if |L|<r and $A^{\prime }\cup L\setminus v$ otherwise. Using Riedl's algorithm, we first note that there is a trailing star centered at 3 with 2 leaves, as seen in Fig. 10. Removing the leaf, there is a trailing star at 1 with 1 leaf. Removing 1 and 2, we have one node left, which is in our $A^{\prime }$. Adding the leaves back and removing 3, we have an $A_{0}$ of 2,3 and 5, a smallest minimal percolating set. Thus, the smallest minimal percolating set with Riedl's algorithm also has size 3, as expected.

To compare with the work of Ref. [15], we shall run the algorithm with F(t)=2 (leading to 2-bootstrap percolation as considered in Ref. [15]) as well as linear-timed function on the following graph:

With our algorithm, we see that nodes 2, 3, and 5 are isolated, respectively, and when we add them to the initial set, all nodes become infected. Thus, the smallest minimal percolating set with our algorithm has size 3.

We shall now compare our algorithm to that of Riedl. A key step in Riedl's algorithm, which is including the leaves of stars and pseudostars in the final minimal percolating set, assumes that these leaves cannot be infected as it is assumed that r>1. However, in our algorithm, we consider functions that may have the value of 1 somewhere in the function, thus we cannot make that assumption. Further, in r-bootstrap percolation, time of infection of each vertex does not need to be taken into account when calculating the conditions for a node to be infected as that r is constant, whereas in the time-dependent case, it is necessary: Suppose a node has n neighbors, and there is only one t such that F(t)≤n, so all neighbors must be infected by time n in order for n to become infected.

The problem our algorithm solves is a generalization of Riedl's, for that it finds one smallest minimal percolating set for functions including constant ones. It has higher computational complexity for that it is not guaranteed for an unisolated node to be infected once one other neighbor of it is infected without accounting for time limits.

## VII. CONCLUDING REMARKS

This paper is dedicated to the introduction and study of a novel time-dependant percolation model. The set up generalises the standard r-bootstrap percolation by introducing a time-dependant percolation function F(t), through which we define *F(t)-bootstrap percolation* (see Definition 1). Some basic properties of F(t)-bootstrap percolation are then studied, with particular attention given to the critical probability $p_{c}$ for certain recurrent functions F(t), for which we give bounds in Sec. II.

Our motivation comes partially from the study of effective vaccination programs which would allow to contain an epidemic, and thus we are interested both in the percolation time of the model, as well as in minimal percolating sets. We study the former in Sec. III, where by considering equivalent functions to F(t), we obtained bounds on the percolating time (see Fig. 2). In particular, the results in Sec. III we show that if F(t)=ℓ is the smallest value the function takes on after some fixed time $t_{0}$, and F(t) has already taken on that value more than times than the number of nodes in the graph, then there will be no nodes that will be infected at that time and the value is safe to be “removed.” The removal process is explained in the same section, and is characterized by obtaining an upper bound on percolation time on a specified tree and function F(t).

In Secs. IV and V we introduce and study smallest minimal percolating sets for F(t)-bootstrap percolation on (nonregular) trees. Our main results appear in Sec. V D, and are given by an algorithm for finding the smallest minimal percolating sets. To show the relevance of our work, we shall conclude this note with a short comparison of our model with those existing in the literature.

Finally, we should mention that the work presented in previous sections could be generalized in several directions and, in particular, we hope to develop a similar algorithm for largest minimal percolating set; and study the size of largest and smallest minimal percolating sets in lattices.

## APPENDIX A: ON NICE FUNCTIONS

In what follows we shall prove Eq. (7). One should note that $F^{\prime }\left(t\right)$ is well-defined. Indeed, since the domain of $F^{\prime }\left(t\right)$ is $I_{2}$, we have that $t\in I_{2}$ and thus $\gamma^{-1}\left(t\right)$ is a valid expression. Moreover, $\gamma^{-1}\left(t\right)$ exists because γ is surjective, and it is unique since $I_{2}$ is an initial segment of N and hence t≠−1. Furthermore, for any $a,b\in I_{1}$, if γ(a)=γ(b)≠−1, then a=b. Since the domain of γ is $I_{1}$, then $\gamma^{-1}\left(t\right)\in I_{1}$. This means that $\gamma^{-1}\left(t\right)$ is in the domain of F(t) and thus one has that $F^{\prime }\left(t\right)$ is defined for all $t\in I_{2}$.

To prove Eq. (7), note that since $\gamma^{-1}\left(0\right)=0$ and the initially infected sets for the models with F(t) and $F^{\prime }\left(t\right)$ are the same, it must be true that $A_{0}^{F^{\prime }}\subseteq A_{0}^{F}$, and in particular, $A_{0}^{F^{\prime }}=A_{0}^{F}=A_{0}.$ To perform the inductive step, suppose that for some $t\in I_{2}$ and $t+1\in I_{2}$, one has $A_{t}^{F^{\prime }}\subseteq A_{\gamma^{-1}\left(t\right)}^{F}$. Moreover, suppose there is a node n such that $n\in A_{t+1}^{F^{\prime }}$ but $n\notin A_{\gamma^{-1}\left(t+1\right)}^{F}$. Then, this means that there exists a neighbor $n^{\prime }$ of n such that $n^{\prime }\in A_{t}^{F^{\prime }}$ but $n^{\prime }\notin A_{\gamma^{-1}\left(t+1\right)-1}^{F}$. Indeed, otherwise this would imply that the set of neighbors of n infected prior to the specified times are the same for both models, and since $F^{\prime }\left(t+1\right)=F\left[\gamma^{-1}\left(t+1\right)\right]$ for $t\in I_{2}$, and thus n would be infected in both or neither models. From the above, since t<t+1 one must have $\gamma^{-1}\left(t\right)<\gamma^{-1}\left(t+1\right)$, and thus

$$\gamma^{-1}\left(t\right)\leq \gamma^{-1}\left(t+1\right)-1.$$

Moreover, since $n^{\prime }\notin A_{\gamma^{-1}\left(t+1\right)-1}^{F}$, then $n^{\prime }\notin A_{\gamma^{-1}\left(t\right)}^{F}$. However, we assumed $n^{\prime }\in A_{t}^{F^{\prime }}$, and since $A_{0}^{F^{\prime }}\subseteq A_{0}^{F}$, we have a contradiction, so it must be true that the sets satisfy $A_{t+1}^{F^{\prime }}\subseteq A_{\gamma^{-1}\left(t+1\right)}^{F}$. Thus, we have proven that for any initially infected set $A_{0}$ and $t\in I_{2}$, one has that Eq. (7) is satisfied for all $t\in I_{2}$.

Through Eq. (7) we can further understand when an F(t)-percolation process finishes in the following manner. Given a percolation function F(t) and a fixed time t∈N, let $t_{p}<t$ be such that $F\left(t_{p}\right)<F\left(t\right)$, and suppose there does not exist another time $t_{i}\in N$ where $t_{p}<t_{i}<t$ such that $F\left(t_{i}\right)<F\left(t\right)$. Suppose further that we use this percolation function on a graph with ℓ vertices. Then, if

$$|\{t_{i}|F\left(t_{i}\right)=F\left(t\right)\}|>\ell ,$$

then there are no nodes that becomes infected at time t. To see this, suppose some node n is infected at time t. Then, this would imply that all nodes are infected before time t. We can show this using contradiction: suppose there exists m nodes $n_{i}$ that there are not infected by time t. Then we know that there exists at least m of $t_{j}\in N$ such that $t_{p}<t_{j}<t$, for which $F\left(t_{j}\right)=F\left(t\right)$ and such that there is no node infected at $t_{j}$. Matching each $n_{i}$ with some $t_{j}$ and letting $t_{k}\in N$ be such that $t_{j}<t_{k}\leq t$, one can see that there is some node infected at $t_{k}$, and $F\left(t_{k}\right)=F\left(t\right)$. Moreover, this implies that there is no $t_{x}\in N$ such that $t_{j}<t_{x}<t_{k}$ and such that there is some node infected at $t_{x}$ and $F(t_{x})=a$. We know such a $t_{k}$ exists because there is a node infected at time t.

From the above, for each $n_{i}$ there are two cases: either the set of nodes infected by $t_{j}$ is the same as the set of nodes infected by $t_{k}$, or there exists node p in the set of nodes infected by $t_{k}$ but not in the set of nodes infected by its $t_{j}$. We have a contradiction for the first case: there must be a node infected at time $t_{j}$ is this is the case, as the set of infected nodes are the same as time $t_{k}$, so the first case is not possible. So the second case must hold for all m of $n_{i}$'s. But then, the second case implies that there is a node infected between $t_{j}$ and $t_{k}$. This means that at least m additional nodes are infected, adding to the at least ℓ−m nodes infected at $t_{i}$ such that $F(t_{i})=a$ and there is a node infected at $t_{i}$, we have at least ℓ−m+m=ℓ nodes infected before t. But if all ℓ nodes are infected before t, this would mean there are no nodes to infect at time t, so n does not exist.

## APPENDIX B: ON EQUIVALENT FUNCTIONS

To show Eq. (8), note that intuitively the function γ constructed above is mapping the index associated to F(t) to the index associated to $F^{\prime }\left(t\right)$. If omitted, then it is mapped to −1 by γ. To prove the statements, we will prove that $P_{F(t)}\left(A\right)=P_{F^{\prime }\left(t\right)}\left(A\right)$. Suppose we have a node n in $P_{F(t)}\left(A\right)$, and it is infected at time $t_{0}$. Suppose $F(t_{0})=a$ for some $a\in Z^{+}$, and let $t_{\text{prev}}$ be the largest integer $t_{\text{prev}}<a$ such that $F(t_{\text{prev}})<a$. Suppose further that $t_{0}$ is the mth instance such that F(t)=a for some t. Moreover, if m>v, then there cannot be any node infected at time $t_{0}$ under F(t), and thus it follows that m≤v. But if m≤v, then $\gamma (t_{0})\neq -1,$ and therefore all nodes that are infected under F(t) became infected at some time t where $\gamma (t_{0})\neq -1$.

Recall that $A_{0}^{F}=A_{0}^{F^{\prime }}$, and suppose for some n such that γ(n)≠−1, one has that $A_{n}^{F}=A_{\gamma (n)}^{F^{\prime }}$. We know that for any $n<t<\gamma^{-1}\left[\gamma \left(n\right)+1\right],\gamma \left(t\right)=-1$, so nothing would be infected under F(t) after time n but before $\gamma^{-1}\left[\gamma \left(n\right)+1\right]$. This means that the set of previously infected nodes at time $\gamma^{-1}\left[\gamma \left(n\right)+1\right]-1$ is the same as the set of nodes infected before time n leading to

$$A_{n}^{F}=A_{\gamma^{-1}\left(\gamma \left(n\right)+1\right)-1}^{F^{\prime }}.$$

Then, since $F\left\{\gamma^{-1}\left[\gamma \left(n\right)+1\right]\right\}=F^{\prime }\left[\gamma \left(n\right)+1\right]$ and the set of previously infected nodes for both are $A_{n}^{F}$, we know that $A_{n+1}^{F}=A_{\gamma (n+1)}^{F^{\prime }}$. Thus, for any time $n^{\prime }$ in the domain of $F^{\prime }\left(t\right)$, there exist a corresponding time n for percolation under F(t) such that the infected set at time n under F(t) and the infected set at time $n^{\prime }$ under $F^{\prime }\left(t\right)$ are the same, and thus $A_{\infty }^{F}=A_{\infty }^{F^{\prime }}$, leading to Eq. (8).
